# Long-term follow-up after retrosternal ileocolic esophagoplasty in two cases of long-gap esophageal atresia: why it is still a valid option as a rescue strategy

**DOI:** 10.3389/fped.2023.1300802

**Published:** 2023-11-22

**Authors:** Matthieu Léonard, Yannick Deswysen, Isabelle Scheers, Maximilien Thoma, Catherine de Magnée, Xavier Stephenne, Dana Dumitriu, Renaud Menten, Raymond Reding, Roberto Tambucci

**Affiliations:** ^1^ Pediatric Surgery and Transplantation Unit, Department of Surgery, Saint-Luc University Clinics, Université Catholique de Louvain, Brussels, Belgium; ^2^ Esophageal, Gastric and Duodenal Surgery Unit, Department of Surgery, Saint-Luc University Clinics, Université Catholique de Louvain, Brussels, Belgium; ^3^ Pediatric Gastroenterology and Hepatology Unit, Department of Pediatrics, Saint-Luc University Clinics, Université Catholique de Louvain, Brussels, Belgium; ^4^ Pediatric Radiology Unit, Department of Radiology, Saint-Luc University Clinics, Université Catholique de Louvain, Brussels, Belgium

**Keywords:** long-gap esophageal atresia, esophageal replacement, ileocolonic transposition, colonic interposition, anti-reflux

## Abstract

**Introduction:**

Esophageal replacement surgery in children is sometimes necessary for long-gap esophageal atresia. Ileocolic esophagoplasty in the retrosternal space can serve as a good alternative technique in case of hostile posterior mediastinum. We present two cases of successful ileocolic transposition performed at 6 months of age.

**Methods:**

Esophageal replacement was performed through a midline laparotomy incision associated with a left cervical approach. The ileocolic transplant was pediculized on the right superior colic artery after ligating the right colic and ileocolic vessels. A retrosternal tunnel was created, and the ileocolic transplant pulled through it to reach the cervical region. Proximally, esophageal-ileal anastomosis and, distally, colonic–gastric anastomosis were performed. Ileocolic continuity was repaired.

**Results:**

There were no early postoperative complications. In both cases, the patients presented oral feeding difficulties during the first 6 postoperative months. Thereafter, full oral feeding was achieved, and both patients were clinically asymptomatic during the following 18 and 20 years, respectively, with satisfactory oral radiological assessments, showing no redundancy or inappropriate growth of the graft and no anastomotic stricture. Currently, these patients do not complain of dysphagia, pathological reflux, or respiratory symptoms.

**Conclusion:**

When native esophagus preservation in long-gap esophageal atresia is estimated unfeasible, ileocolic transposition in the retrosternal space might be considered a good and safe option, particularly in those difficult cases after multiple previous surgical attempts and mediastinitis. This technique is putatively associated with a beneficial anti-reflux effect, thanks to the presence of the ileocecal valve, in preventing cervical peptic esophagitis. Long-term follow-up confirms that the transposed colon in the retrosternal space did not suffer any abnormal modification in size and growth.

## Introduction

Long-gap esophageal atresia represents one of the most challenging and debated topics in pediatric surgery (1). Although several consensus, conferences, and position papers stated that efforts should be made to preserve native esophagus, this is sometimes ultimately not feasible (2, 3). Accordingly, some form of esophagoplasty is required to provide orogastric continuity. Surgeons are faced with various choices, including different conduits, such as the stomach, colon, and jejunum, and various routes, each carrying its own set of advantages and disadvantages (4, 5). Currently, orthotopic placement in the posterior mediastinum is the most common strategy, combined with either gastric pull-up or colonic replacement (1, 2). Alternatively, a retrosternal route can be considered to avoid any intra-thoracic approach, particularly after previous surgical attempts and mediastinitis (6). In this context, ileocolic esophagoplasty might constitute an alternative to obtaining enough length to allow tension-free anastomosis (7). This technique is also putatively associated with a beneficial anti-reflux effect related to the presence of the ileocecal valve, which might prevent cervical peptic esophagitis (8, 9).

We report the surgical strategy and long-term outcomes in two patients with long-gap esophageal atresia who underwent esophageal replacement using retrosternal ileocolonic segment during early infancy.

## Materials and methods

### Surgical technique

**Figure 1:**
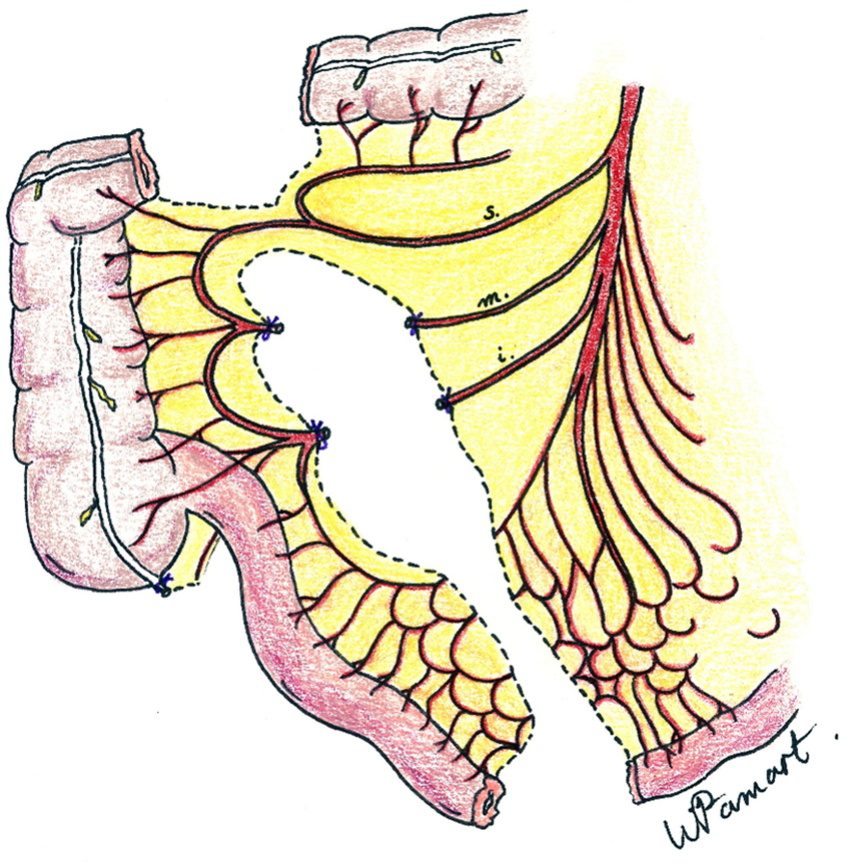
Vascular supply of ileocolic conduit showing preservation of the right superior colic artery (s = right superior colic artery; m = right middle colic artery; I = ileoceco-appendicular artery).

Under general anesthesia, the patient is placed in the supine position, with the neck hyperextended and the head turned to the right side. First, the peritoneal cavity is entered through a midline incision from the xiphoid to below the umbilicus. The terminal ileum, cecum, and ascending colon are mobilized. The arterial supply of the right colon is inspected. The base of the ileocolic, right colic, and ileal arteries are exposed. Atraumatic vascular clamps are placed on these arteries, and the viability of this segment of the colon, now supplied by the right superior colic artery, is assessed. The transplant is ultimately pediculized on the right superior colic artery after interruption of the right middle colic and ileocolic vessels (Figure 1). The length of the colon for esophageal replacement is estimated. The ileum is transected 5–10 cm proximally to the ileocecal junction and the transverse colon distally, keeping the right superior colic artery with the plasty. Intestinal continuity is restored by end-to-end anastomosis between the terminal ileum and the right transverse colon. The mesenteric defect is closed to prevent bowel herniation. The inferior esophageal stump is individualized from the posterior vagus nerve and resected, and the stomach is closed using a running suture. The diaphragmatic hiatus is closed by resorbable stiches. The attachments of the diaphragm to the xiphoid process are dissected, and a retrosternal tunnel is meticulously fashioned through blunt finger manipulation, all without the need to open the thoracic cavity. It is essential that this tunnel is sufficiently spacious to accommodate the plasty without exerting excessive pressure on its blood supply.

**Figure 2:**
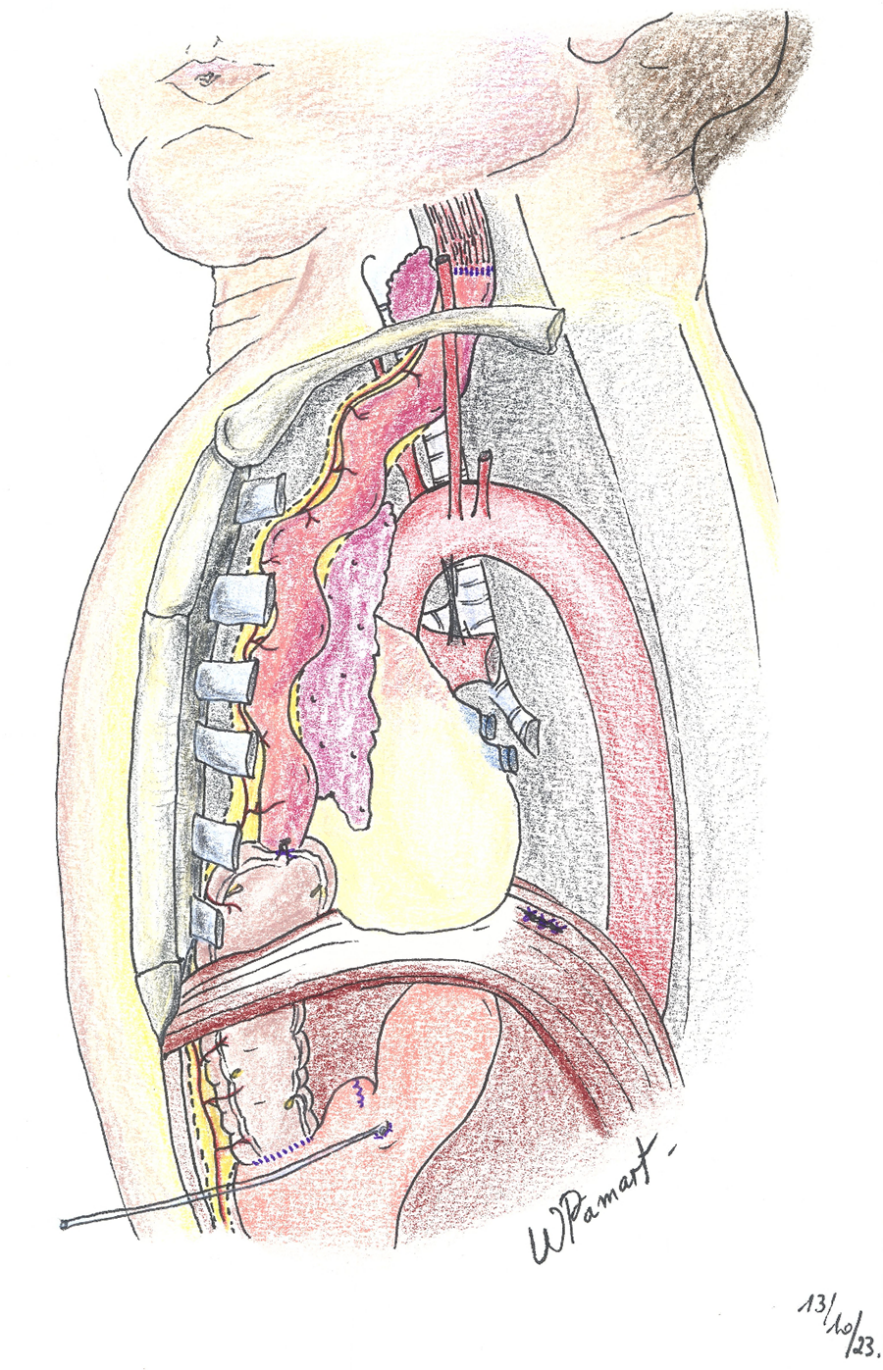
Lateral view illustrating the retrosternal route.

**Figure 3:**
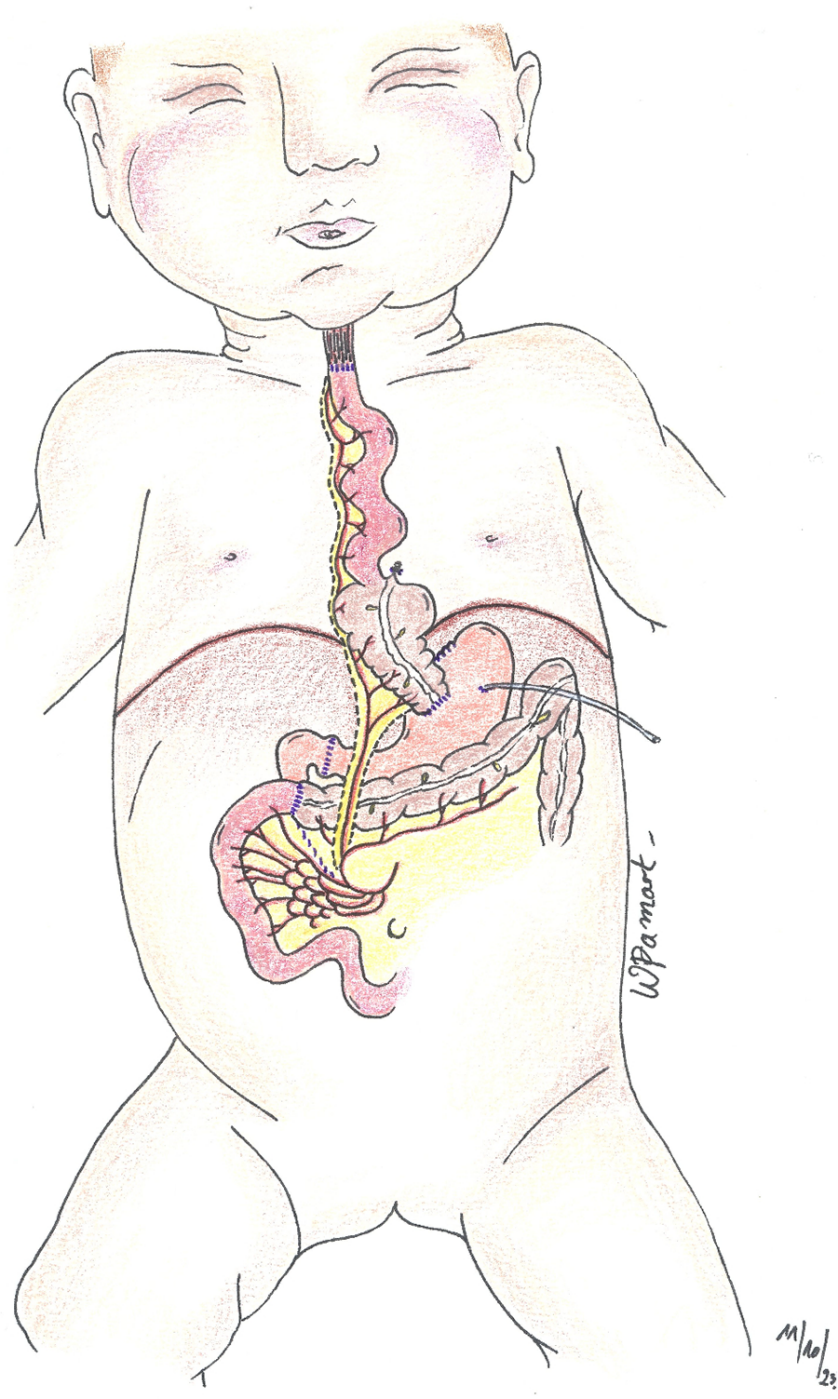
Anterior view of ileocolic plasty after anastomosis.

A left cervical incision is performed following the edge of the anterior part of the sternocleidomastoid muscle. The platysma and the sternal head of the sternocleidomastoid muscle are cut. The deeper sternohyoid and sternothyroid muscles are divided to visualize the lateral edge of the thyroid. The internal jugular vein and the carotid artery are left externally. The trachea and esophagus are identified. Retrosternal space is created using fingers and blunt dissection. Such space is entered from below the sternocleidomastoid muscle, and the superior portion of the tunnel is enlarged enough to prevent compression of the ileocolic pedicle within the anterior mediastinum or at the thoracic inlet. The ileocolic graft with its vascular pedicle is brought up in the front of the stomach and placed in the retrosternal space in an iso-peristaltic fashion (Figure 2). The ileal stump is anastomosed to the lower part of the proximal native esophagus in an end-to-end anastomosis using a resorbable suture. The distal portion of the colon is anastomosed to the anterior wall of the stomach (Figure 3). The anastomosis is performed using a double-layered resorbable suture. A high location on the cardia is necessary to prevent air trapping and bloating. If the gastrostomy is located at the cologastrostomy site, it is carefully removed. The gastrostomy tube is then repositioned proximally to the cologastrostomy site and directed out through the initial stab wound opening in the abdominal wall. Pyloroplasty (Heineke–Mikulicz) is performed routinely. Surgical wounds on the neck and the abdomen are sutured layer by layer.

### Case report 1

A 38-week gestational age newborn girl with a birth weight of 2.3 kg was diagnosed with type A long-gap pure esophageal atresia according to Gross classification, which was suspected prenatally. At birth, a Replogle double-lumen tube was placed for continuous saliva suction, and a gastrostomy was created by mini-laparotomy. A VACTERL workup excluded associated anomalies. Esophagogastric radiography with opacification through the gastrostomy showed type A esophageal atresia with a gap of 4.5 cm.

At the age of 5 months, ileocolic transposition in the retrosternal space was performed according to the surgical technique described above.

Upper gastrointestinal contrast study was performed on postoperative day 6, confirming the absence of any anastomotic leakage. Accordingly, oral feed was started. She was discharged home on postoperative day 14. Full oral feeding was achieved 4 months after the operation, and the gastrostomy could be removed after 2 months.

**Figure 4:**
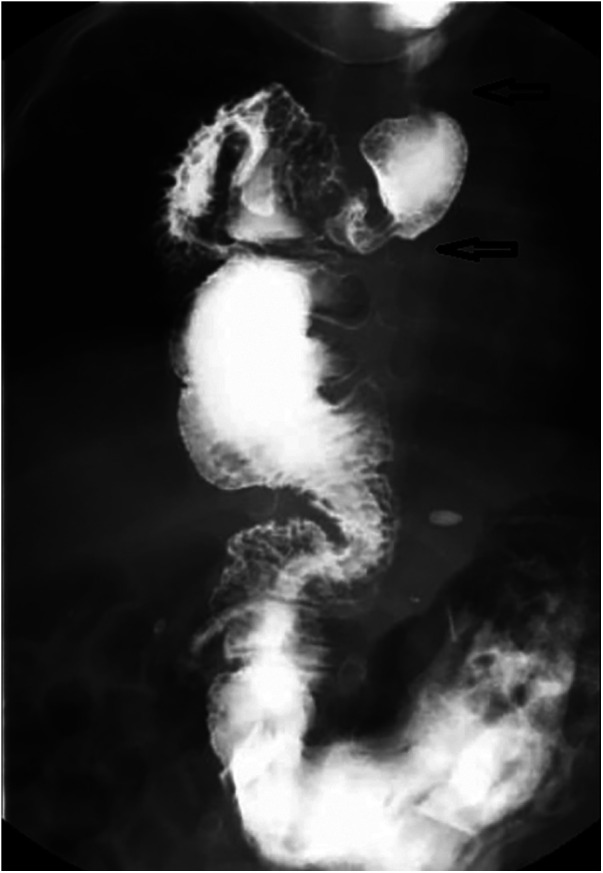
Case 1: upper gastrointestinal series performed at the age of 6 months (1-month post-surgery) showing esophagoileal anastomosis (upper black arrow) and ileocecal valve (lower black arrow).

A further contrast study with Barium ingestion at 6 months showed no stricture (Figure 4).

**Figure 5:**
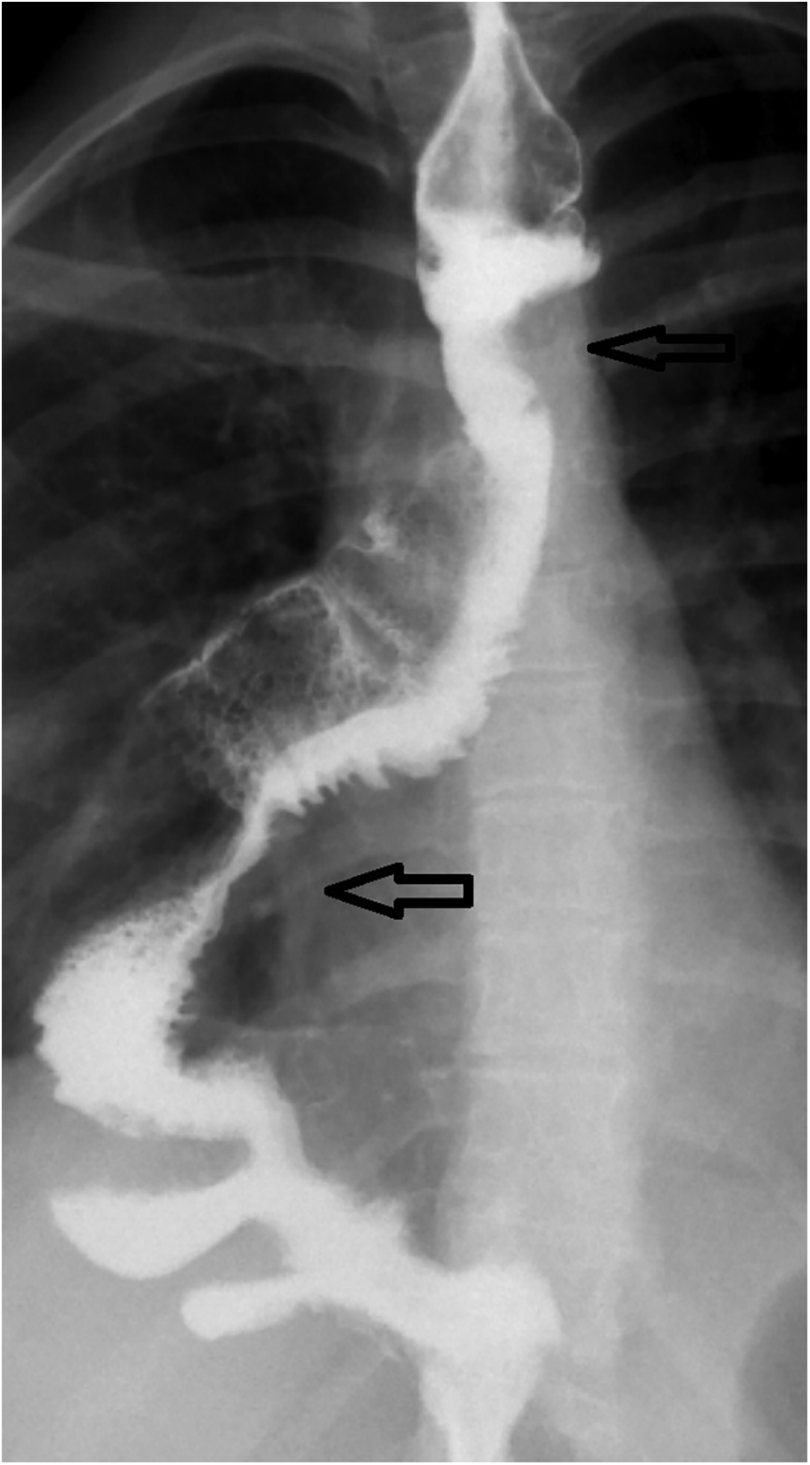
Case 1: upper gastrointestinal series performed at the age of 18 years demonstrating the esophagoileal anastomosis (upper black arrow) and ileocecal valve (lower black arrow).

The patient evolved well afterward. The last assessment was performed at the age of 18 years. The patient has no dysphagia and no signs of pathological reflux in the esophageal remnant, confirmed by routine endoscopy studies. She did not complain of respiratory diseases, including recurrent aspiration or pneumonia. The last contrast study, 1 year ago, showed that the graft exhibited appropriate growth without colonic dilatation or stenosis (Figure 5).

### Case report 2

A female infant with a birth weight of 2.5 kg was born at 36 weeks gestational age and diagnosed with VACTERL association. On the first day of life, in her country of origin, type A long-gap esophageal atresia was found, and a right thoracotomy was performed. In this situation, a right cervical esophagostomy and a gastrostomy were created. Twenty days later, a sigmoidostomy was performed due to an anorectal malformation (rectovestibular fistula). The child was thereafter referred to our Institution, where an ileocecal esophagoplasty in the retrosternal space and a posterior sagittal anorectoplasty (PSARP) were simultaneously performed at the age of 6 months. During the surgery, gastrostomy was closed since it was created at the site of the future cologastric anastomosis. The first postoperative month was marked by oral feeding difficulties. Accordingly, a new gastrostomy was created 2 months later to allow gastric feeding without a nasogastric tube, and sigmoid continuity was restored in the same operative time. Subsequently, oral feeding was progressively fully achieved, and the gastrostomy was removed 6 months later. The patient was followed yearly until the age of 6 years and once again at 12 years through radiologic and endoscopic assessments. Then, she was followed remotely, and currently, at the age of 20 years, the patient denies dysphagia and other digestive or respiratory complaints.

## Discussion

According to the ERNICA (European Reference Network for rare Inherited and Congenital Anomalies) consensus conference, no agreement on the optimal approach for esophageal reconstruction in case of long-gap esophageal atresia has been reached (2). No randomized controlled trials have been conducted to compare surgical repair methods or techniques (2). It is important to emphasize that the preservation of the patient's esophagus should be prioritized before considering any alternative technique, as no other conduit can adequately fulfill its function of transporting food from the oral cavity to the stomach (3).

In the past few years, there has been a growing popularity in using esophageal traction-and-growth strategies (10). External traction (Foker technique) and extra-thoracic esophageal elongation (Kimura procedure) were proposed several years ago; their use has now gained in popularity in the field, and the combination of both techniques has been reported in very few cases (11–13). More recently, a technique using staged thoracoscopic internal traction has emerged and has demonstrated encouraging outcomes (14). Such strategies can now be performed even thoracoscopically, possibly shortly after birth, as suggested by certain authors, even avoiding the placement of a gastrostomy (15). Magnetic compression anastomosis has been recently published again, possibly in combination with mini-invasive surgery (16).

Sometimes, the esophageal segments may fail to achieve apposition, requiring further efforts, including the use of different types of mucosal-muscular flaps, circular or spiral myotomies, and elongation of the lesser gastric curvature (17–22). If primary esophageal anastomosis is not feasible, esophageal replacement techniques must be considered using jejunal, gastric, or colonic grafts (2).

As recommended by the INoEA (International Network of Esophageal Atresia) working group, jejunal interposition is preferred among these techniques due to its similar growth rate to the child and its preservation of intrinsic motility (1, 23). Furthermore, it carries a lower risk of gastroesophageal reflux, which can potentially lead to long-term pulmonary complications compared to gastric pull-up and colonic replacement (23). Two methods of jejunal interposition are employed, including using the jejunum on its pedicle or a free jejunal graft with microvascular anastomoses (24, 25). Jejunal interposition is an infrequently employed approach due to technical challenges. However, it yields favorable outcomes in centers with specialized expertise (7).

Recent investigations, including a survey of European pediatric surgeons and a comprehensive review by Von Allmen et al., reported that when an esophageal replacement was deemed necessary, the procedure of choice was gastric pull-up (26, 27). Gastric transposition offers several benefits, such as an excellent blood supply and a simple procedure with a single anastomosis requirement (13, 28). In a study encompassing more than 200 cases documented by Lewis Spitz, the procedure exhibited a mortality rate of approximately 5%. Moreover, the significance of morbidity remains noteworthy, with reported issues including anastomotic leaks, anastomotic strictures, and swallowing difficulties (29). Nevertheless, gastric transposition demonstrates favorable outcomes in clinical follow-up, including adequate oral feeding, normal social life, and regular growth (30). An alternative method utilizing the stomach is the gastric tube, using the outer curvature of the stomach, either isoperistalticly or anisoperistaltic, which has been described in very few reports (7, 29, 31). Gastric pull-up is currently performed transhiatally, possibly with a laparoscopic and cervical approach, avoiding any thoracotomy. This strategy provides several advantages, but it has a limited employment in case of severe scars in the posterior mediastinum due to previous surgical attempts and mediastinitis.

The INoEA working group noted that colonic interposition is predominantly reserved as an ultimate measure to be employed when all other techniques have been exhausted or are deemed unfeasible (1). However, based on the literature review by Sharma and Gupta and a retrospective study by Lima et al., the colon stands out as the favored and safest option for esophageal replacement (7, 32). This distinction is attributed to its relative safety, simplicity in execution, and notably diminished incidence of infrequent and severe complications compared to alternative techniques (7). Kelling and Lundblad were the first to describe colonic replacement for esophageal substitution (33, 34). Colon replacement can be performed utilizing the right, left, or transverse colon, determined by the vascular pedicle, which represents a crucial technical consideration influencing short-term complications in this procedure (35, 36). The utilization of the right colon and transverse colon as anti-peristaltic conduits, relying on the vascular supply from the middle colic artery, contrasts with the deployment of the transverse or left colon as an iso-peristaltic conduit, supported by the left colic vessels. This preference for the latter is attributed to its diminished bulkiness and more dependable vascular perfusion than the right colon (37, 38). Nevertheless, colon replacement can lead to various complications, including the development of kinking due to inappropriate growth, protrusion of the graft in the neck, food impaction within the graft leading to reflux, and the risk of both aspiration and gastroesophageal reflux (39).

In the case of replacement, one of the most common long-term complications is reflux gastritis in gastric pull-up and gastrocolic reflux in colonic replacement (7, 29). In this context, the theory of the ileocecal valve as an anti-reflux mechanism in ileocolic plasty emerged. This technique was first described in 1953 by Javid in Chicago, where a substernal ileocolic interposition was successfully performed in an 18-month-old child with isolated esophageal atresia (8). Subsequently, pediatric surgeons adapted this technique to treat esophageal atresia and strictures. In 1994, Touloukian published the first series of eight patients who underwent ileocolic plasty with a follow-up of up to 10 years postoperatively (9). In 1996, Raffensperger reported a series of 48 patients who underwent esophageal reconstruction using this procedure, with a follow-up period ranging from 1 to 37 years (27). In the Touloukian series, none of the patients displayed postoperative respiratory complications or symptomatic gastroesophageal reflux. In the Raffensperger series, only one patient demonstrated gastrocolic reflux and recurrent pneumonia. These results confirm a potential anti-reflux role of the ileocecal valve. These two series have shown some other benefits of performing ileocolic plasty. The ileum maintains excellent peristalsis, efficiently propelling food (40). In addition, retaining the ileocecal valve may reduce the malodorous breath associated with food stagnation in a redundant colon (9). Moreover, there is a better size match between the esophagus and terminal ileum, allowing for an end-to-end anastomosis (40). Furthermore, the presence of a constant, wide mesenteric gap between the ileal and colic branches or the ileocolic vessel enables mobilization of the ileum to achieve the desired length (41). Nevertheless, ischemic necrosis of the transplanted segment remains the most severe immediate complication (40). Also, it has been reported that following ileocolic plasty, the lack of function of the distal ileum might lead to metabolic disorders. However, no deficiencies in Vitamin B12 or fat absorption impairment have been described in the literature. The literature has sparsely described the utilization of ileocolic plasty in the case of long-gap esophageal atresia. In a recent study, Lima et al. conducted a retrospective analysis of the outcomes of esophagocoloplasty in children, with a follow-up period extending up to 45 years. Within this series, 11 patients underwent ileocolic esophageal replacement, yielding favorable outcomes in terms of overall quality of life (32). Bal and Sen reported encouraging outcomes in their series involving six children who underwent ileocolic esophageal replacement, with an average follow-up period of 37 months (41). Remarkably, all six children remained free of dysphagia throughout the follow-up.

Another aspect of interest regarding the surgical technique described is the retrosternal route. There are essentially two main approaches for positioning the organ within the chest: substernal (retrosternal) and mediastinal (transhiatal, native, orthotopic) (7). The retrosternal route offers advantages such as technical simplicity, safety, and ease of implementation, especially in pediatric cases. Additionally, this approach has been recommended to lower the risk of postoperative pneumonia. This phenomenon arises because the inflation of the stomach or colon with air and/or fluid in the early postoperative period does not exert pressure on the lungs, as opposed to be associated with the mediastinal route (42, 43). The posterior mediastinal pathway is linked to pronounced tachyarrhythmias, inappropriate sinus tachycardia, bradycardia, and postoperative hypotension. Similar observations have been documented in medical literature and are thought to be connected to autonomic instability due to the proximity of the vagal and sympathetic nerves to the repositioned stomach within the posterior mediastinum, direct manipulation of the atrium or pericardium during mediastinal dissection and manipulation, as well as the hyperadrenergic state following surgery (42). Importantly, the retrosternal route avoids the need for a thoracotomy and its pulmonary complication risks (9). Accordingly, the retrosternal approach gains increased relevance, particularly when dealing with a scarred mediastinum due to previous explorations, as observed in cases of previous surgical attempts, particularly after anastomotic leakage and consequent mediastinitis (7). In such situations, access to the posterior mediastinum is sometimes unfeasible and/or associated with pulmonary parenchymal injuries, which can subsequently lead to pleural infections and/or bronchial-esophageal fistulas. It has to be noted that the retrosternal approach, as described above, has limitations or contraindications in the case of previous sternotomy, anterior scars, as well as after anterior aortopexy.

However, the retrosternal route presents a higher occurrence of anastomotic stenosis when compared to the posterior mediastinal route (44). This complication could be attributed to the graft's compression at the thoracic inlet (45, 46). To mitigate this possibility, some authors opt for a partial resection of the *manubrium sterni* and clavicle (47). In a recent meta-analysis conducted by Booka et al. on an adult series, anastomotic leakage was significantly reduced when the posterior mediastinal route was used, while the occurrence of pneumonia was significantly lower in those cases where the retrosternal route was employed. Furthermore, this review indicated that there was no significant disparity in mortality rates between the retrosternal and posterior mediastinal routes (48).

In conclusion, native esophageal preservation should be considered the first option, even after previous surgeries and in case of a very long gap (11). Nonetheless, the patients reported above experienced a favorable postoperative recovery, a fast full oral feeding, and a long-term follow-up characterized by the absence of dysphagia, recurrent respiratory complaints, or redundant growth of the graft, demonstrating that this technique presented notable results. Accordingly, we suggest that this technique might be considered in those complicated cases in which, after previous surgeries and infections, the mediastinum is estimated or proven to be hostile due to severe scars. When a retrosternal route is preferred, the use of the distal ileum and right colon easily allows us to reach the neck. This technique is also putatively associated with a beneficial anti-reflux effect, thanks to the presence of the ileocecal valve, in preventing cervical peptic esophagitis. Long-term follow-up is recommended until adulthood, followed by subsequent transition to adult care.

## Data Availability

The original contributions presented in the study are included in the article/Supplementary Material, further inquiries can be directed to the corresponding author.
